# Pro-regenerative Dialogue Between Macrophages and Mesenchymal Stem/Stromal Cells in Osteoarthritis

**DOI:** 10.3389/fcell.2021.718938

**Published:** 2021-09-17

**Authors:** Candice Bohaud, Rafael Contreras-Lopez, Jholy De La Cruz, Claudia Terraza-Aguirre, Mingxing Wei, Farida Djouad, Christian Jorgensen

**Affiliations:** ^1^IRMB, Univ Montpellier, INSERM, Montpellier, France; ^2^CellVax, Villejuif, France; ^3^CHU Montpellier, Montpellier, France

**Keywords:** osteoarthritis, therapy, regeneration, mesenchymal stromal/stem cells, macrophages

## Abstract

Osteoarthritis (OA), the most common degenerative and inflammatory joint disorder, is multifaceted. Indeed, OA characteristics include cartilage degradation, osteophytes formation, subchondral bone changes, and synovium inflammation. The difficulty in discovering new efficient treatments for OA patients up to now comes from the adoption of monotherapy approaches targeting either joint tissue repair/catabolism or inflammation to address the diverse components of OA. When satisfactory, these approaches only provide short-term beneficial effects, since they only result in the repair and not the full structural and functional reconstitution of the damaged tissues. In the present review, we will briefly discuss the current therapeutic approaches used to repair the damaged OA cartilage. We will highlight the results obtained with cell-based products in clinical trials and demonstrate how the current strategies result in articular cartilage repair showing restricted early-stage clinical improvements. In order to identify novel therapeutic targets and provide to OA patients long-term clinical benefits, herein, we will review the basis of the regenerative process. We will focus on macrophages and their ambivalent roles in OA development and tissue regeneration, and review the therapeutic strategies to target the macrophage response and favor regeneration in OA.

## Introduction

Osteoarthritis (OA) is the most common degenerative and inflammatory joint disorder. Although the prevalence of OA is continuously increasing, so far no biological or pharmacological therapy exists to both control inflammation and restore joint tissue integrity. Thus, OA is still an incurable condition with only palliative treatments to alleviate pain and joint replacement with prosthesis as ultimate option.

The main OA alterations appear progressively over time without a particular defined chronological order and include cartilage damage, osteophyte formation, subchondral bone remodeling, and a chronic low-grade inflammation (Chen et al., 2017). Cartilage homeostasis is insured by articular chondrocytes, which are quiescent and differentiated cells that maintain the balance between the catabolic and anabolic functions in healthy cartilage. In OA, chondrocytes respond to deleterious stresses by undergoing intrinsic modifications such as an abnormal production of the extracellular matrix (ECM) and an increased activity of proteolytic enzymes. Moreover, chondrocyte apoptosis clearly occurs in OA cartilage. Since chondrocytes exhibit a limited proliferation rate (Hwang and Kim, 2015), articular cartilage possesses a restricted repair potential, resulting into a progressive cartilage degradation and loss when damaged.

Repair or regeneration of an injured tissue or organ occurs when the tissue is damaged or removed. While tissue regeneration consists in the structural and functional reconstitution of the damaged/removed tissue or organ, tissue repair results in the wound healing by fibrosis and scar formation. Although giving rise to different outcomes, these two processes trigger an inflammation phase and end with a resolution phase. However, one of the main distinctions between what drives either regeneration or repair is the inflammatory response following tissue damage or removal. The inflammatory response in regenerative mammalian models is characterized by massive but transient accumulation and activation of innate immune cells including neutrophils, monocytes, and macrophages (Cowin et al., 1998). The primary goal of inflammation is to protect the damaged tissue against foreign bodies (Oishi and Manabe, 2018). More recent studies have shown that beyond this protective role, inflammation has also a functional role during the regeneration process (Hasegawa et al., 2017; Nguyen-Chi et al., 2017). Indeed, proper levels of tumor necrosis factor alpha (TNF-α) (Nguyen-Chi et al., 2017) and interleukin-1β (IL-1β) (Hasegawa et al., 2017) produced by myeloid cells and in particular macrophages are of paramount importance for regenerative cell survival, proliferation and regeneration.

In contrast, an abnormal macrophage response characterized by an uncontrolled release of cytokines, chemokines, and cartilage-degrading enzymes has been shown to be responsible for OA development and progression (Kraus et al., 2016; Sarmanova et al., 2016; Raghu et al., 2017). Indeed, synovial macrophages have been shown to be pivotal in the OA vicious circle of cartilage degradation and inflammation (Sambamurthy et al., 2018).

In the present review, we will focus on the ambivalent role of macrophages in OA tissue degradation, repair, and regeneration. The identification of the mechanisms underlying the tight regulation of the inflammation phase during tissue regeneration that could be applied to degenerative diseases such as OA is of major importance in the field of regenerative medicine.

### Osteoarthritis: From the Clinical Problem to the Current Therapeutic Strategies

According to an initial paradigm, OA was considered as the consequence of a tear and wear process responsible for cartilage degradation, and the production of osteophytes suggested to be a bone response process to protect and stabilize the damaged joint (Felson, 2013; Berenbaum et al., 2017). OA has been proposed as a whole joint disease. Indeed, OA is a heterogeneous multifaceted disorder with cardinal alterations that appear progressively to varying degrees and that comprises cartilage lesions, subchondral bone remodeling, osteophyte formation and a chronic low-grade inflammation, ligaments breakdown, and loss of normal joint function (Kraus et al., 2015; Chen et al., 2017).

The risk factors for OA development are multiple. This risk increases with age and in particular in women. Genetic predispositions, bone deformities and certain metabolic disorders, such as diabetes and hemochromatosis, have been also incriminated in OA pathogenesis. Among the other risk factors, joint injuries that occur during sport activities and accident increase the risk to develop OA. A decade ago, weight or body mass index were also associated with the development of hand OA. Indeed, compared to a non-obese population, epidemiological studies showed that obese individuals exhibit a twofold increased rate of hand OA (Yusuf et al., 2010). This was explained by the systemic release of pro-inflammatory cytokines (adipokines) by the abdominal adipose tissue and the intraarticular fat pads responsible for a low-grade inflammation susceptible to affect peripheral tissues such as joint tissues (Berenbaum, 2013; Ioan-Facsinay and Kloppenburg, 2013). Matrix metalloproteinases (MMP) and a disintegrin and metalloproteinase with thrombospondin motifs (ADAMTS) enzymes associated with cartilage degradation and regulated by pro-inflammatory cytokines have been incriminated in OA pathogenesis. Moreover, synovial membrane inflammation, occurring both in the early and late phases of OA, was shown to be related to alterations in the adjacent cartilage such as in rheumatoid arthritis. The defective repair process of the whole damaged joint compartments that occurs under different circumstances is responsible for the joint tissue changes and inevitably OA. The progression rate of OA varies over time according to the patients, resulting in different signs and symptoms such as pain and reduced motion ability.

In addition to macrophages, several immune cells including T cells, B cells, mast cells, natural killer cells, granulocytes, and dendritic cells infiltrate the synovial membranes of OA patients. While macrophages are the most abundant immune cells within the OA synovium with approximately 65%, 20% are T cells (Li et al., 2017). Although several studies have clearly demonstrated the accumulation of different T cells subsets such as T helper (Th) 1, Th2, Th9, Th17, Th22, and regulatory T cells in the peripheral blood, synovial fluid or tissue of OA patients, their exact roles in the pathogenesis of OA are poorly known (Li et al., 2017; Weber et al., 2019). In particular, little is understood on the influence of the immune system dysregulation in OA on osteoblasts and subchondral bone disturbance (Weber et al., 2019). Therefore, further studies are needed to better understand the role of osteoimmunology dysfunction on OA pathogenesis and how this dysfunction might be linked to the decline in mesenchymal stromal cell number and “fitness” in the bone marrow niche (Ganguly et al., 2017) to identify novel therapeutic targets in OA management.

Articular chondrocytes, pivotal for cartilage homeostasis by maintaining the balance between anabolic and catabolic functions in healthy cartilage, respond to deleterious biochemical and biomechanical stresses in OA by undergoing phenotypic and functional changes that include an abnormal ECM production and an increase in extracellular proteolytic enzyme activity. Macrophages localized in the synovial membrane have been shown to be pivotal in the vicious circle of OA cartilage degradation and inflammation (Sambamurthy et al., 2018). The debris resulting from the cartilage breakdown stimulate macrophages of the inflamed synovial membrane and create a vicious circle promoting their capacities to release catabolic factors and pro-inflammatory cytokines (Chen et al., 2020). Moreover, macrophage-mediated inflammatory response was also proposed to be fatty acid binding protein 4 (FABP4) dependent. Indeed, macrophages also express adipokines such as FABP4, highly expressed by adipocytes to facilitate the lipid transportation to specific cell compartments. The genetic or pharmaceutical inhibition of FABP4, associated with obesity and metabolic diseases, significantly reduced synovium hypertrophy and the infiltration of macrophages at early OA stage in mice fed with high-fat diet (Zhang C. et al., 2018). Therefore, the association between adipokines and macrophage-mediated inflammatory response might link obesity and metabolic diseases with OA development and progression.

Considering the pathobiological heterogeneity and the diversity of OA characteristics from one patient to another, all the disease aspects including inflammation and joint tissue degradation should be considered to apply the most appropriate and personalized OA therapy at an early stage of the disease to avoid massive and irreversible structural and functional alterations. Currently, there is a lack of strategy for OA patient diagnosis and stratification, which makes impossible the implementation of a personalized therapeutic strategy. In this context, although promising therapies have emerged in the advanced disease stage, none of them has been proven to positively and significantly change the disease progression or successfully prevent final joint replacement.

Among the current promising approaches, we can discriminate pharmacological and stem cell therapies. Some of the drugs that change OA pathophysiology and reduce the structural damage to limit long-term disability, also referred as disease-modifying OA drugs (DMOAD), are undergoing phase-2 and phase-3 clinical trials (Oo and Hunter, 2019; Oo et al., 2021). While OA is a multifaceted disease, DMOAD will target mainly one aspect of the disease at a time such as cartilage anabolism and catabolism, inflammatory responses, subchondral bone or pain processes. However, in the OA vicious circle of cartilage degradation and inflammation, treatments that target cartilage might also have consequences on the inflammatory processes and vice versa. The most promising anabolic DMOAD is the fibroblast growth factor (FGF)-18 studied in a phase-2 multicenter randomized clinical study. Indeed, the intra-articular (IA) administration of 100 μg sprifermin, a truncated product of recombinant human FGF18 (rhFGF18), every 6 or 12 months led to an amelioration in total femorotibial joint cartilage thickness after 2 years in OA patients (Hochberg et al., 2019). Although promising, since it is the first molecule that demonstrates a structural effect on quantitative MRI and radiography, the sprifermin failed at providing a symptomatic effect. Indeed, no difference in the symptoms and, in particular, on the WOMAC has been reported between the sprifermin and placebo groups. Therefore, further clinical studies on FGF18 are required, alone or in combination with other therapeutics, with longer times of observation and more in-depth analyses on the joint structure and function. Of note, while some studies report beneficial effect of DMOAD with anabolic effect such as sprifermin on cartilage, none have been reported with anticatabolic factors including MMP inhibitors such as CP-544439, AZD-8955, or PG-530742 (Karsdal et al., 2016).

Regarding DMOAD with anti-inflammatory effects, treatment targeting IL-1 and TNF-α have been used in clinical trials with minimal or no clinical benefits (Grassel and Muschter, 2020). Indeed, among the molecules that repress the IL-1 activity, Anakinra administration has shown no improvement of OA symptoms and AMG108 and Lutikizumab have shown minimal clinical benefit (Cohen et al., 2011) or effect on WOMAC pain score (Fleischmann et al., 2019). Regarding anti-TNF-α agents, Adalimumab and Etanercept failed in reducing OA patient pain (Aitken et al., 2018; Kloppenburg et al., 2018), and Infliximab reduced hand OA progression (Loef et al., 2018). Similarly, diacerein, a purified anthraquinone derivative with a repressive effect on the production of IL-1 and metalloproteases production, has been studied (Dougados et al., 2001). In a review including 2,210 patients from 10 clinical trials, only a minimal symptomatic effect was reported with that drug as compared with placebo (Fidelix et al., 2014). Diacerein has been proposed to be beneficial for OA patients with contraindication to NSAID or paracetamol, since its efficacy was shown to be equivalent to that of non-steroidal anti-inflammatory drugs (NSAIDs) and better than paracetamol (Pavelka et al., 2016). However, no drugs are currently approved as OA disease-modifying agents. Therefore, a shift from single- to multitarget non-pharmacological therapies, thought to relieve symptoms in the long term and to limit functional loss, has been seen.

In this context, mesenchymal stem/stromal cell (MSC)-based therapy represents a promising approach since MSC exhibit regenerative properties through the secretion of bioactive factors that have potent cytoprotective, antiapoptotic, antifibrotic, and anti-inflammatory effects (Le Blanc and Ringden, 2006; Djouad et al., 2009; Caplan and Correa, 2011; Maumus et al., 2013). Thus, based on their biological properties and results obtained after intra-articular (IA) injection of murine MSC in experimental OA showing a reduced synovial thickening, osteophyte formation, and cartilage destruction (ter Huurne et al., 2012; Diekman et al., 2013; Schelbergen et al., 2014), IA injection of MSC from various sources (bone marrow and adipose tissue) in OA patients was contemplated. A large number of clinical trials has thus been initiated and has shown that IA MSC injection is well-tolerated and exhibited promising clinical results. Indeed, a recent meta-analysis of randomized controlled trials revealed that MSC administration in patients with knee OA significantly decreased the pain and improved the function and the stiffness as compared with control (Qu and Sun, 2021). However, this conclusion drawn from nine randomized controlled trials including three with small sample size needs to be confirmed with larger-scale randomized controlled trials in order to draw a more robust conclusion on MSC efficacy to treat patients with knee OA. Moreover, although promising MSC administration in OA patients does not allow consistent long-term beneficial effects mainly because MSC improve OA development and progression by regulating the immune response without promoting joint tissue regeneration (Luz-Crawford et al., 2016; Pers et al., 2018). Therefore, a better understanding of the mechanisms that allow tissue restoration in regenerative models might be of interest to bring innovative treatments to OA patients.

### Tissue Inflammation and Regeneration

Regeneration in small vertebrate organisms or in adult mammalian tissues/organs is orchestrated by the immune response (Wynn and Vannella, 2016; Laplace-Builhe et al., 2020). Immune cells including macrophages are recruited at the site of injury and actively participate to the formation of new tissues, organs, or limbs (Wynn and Vannella, 2016; Laplace-Builhe et al., 2020). Depletion of these cells leads, regardless of the study model, to alterations in tissue restoration (Duffield et al., 2005; Summan et al., 2006). Thanks to their high plasticity, macrophages adopt different phenotypes referred as pro- or anti-inflammatory macrophages to simplify and thus direct the two phases of regeneration: inflammation and its resolution (Arnold et al., 2007). The disruption of this cellular response and of these finely regulated phases lead to regeneration defects (Wynn and Vannella, 2016).

#### Macrophages in Mammalian Muscle Regeneration

An interesting regenerative model in mammals is the skeletal muscle, which can regenerate completely after minor injuries. Muscle stem cells, called satellite cells, are able to differentiate into myoblasts, which will become myocytes and form new muscle fibers after injury (Sciorati et al., 2016; Baghdadi and Tajbakhsh, 2018; Wosczyna et al., 2018). Macrophages actively participate in the formation of these new muscle structures, since their depletion inhibits the regeneration of this tissue, but their role is not yet fully established (Przybyla et al., 2006; Summan et al., 2006; Arnold et al., 2007; Segawa et al., 2008; Melton et al., 2016). In adult tissues, macrophages can emerge from two sources. One main source of macrophages is a subset of circulating monocytes derived from bone marrow hematopoietic stem cells, which are recruited to wound sites after injury, where they differentiate into mature macrophages (Hoeffel and Ginhoux, 2018). The other one consists in a subset of erythro-myeloid progenitor cells that emerges during embryo development, giving rise to tissue-resident macrophages, which are capable of self-renewal and are reportedly responsible for tissue homeostasis and persist during the adulthood (Hoeffel and Ginhoux, 2018; Wu et al., 2020). When the tissue is damaged, circulating monocytes are recruited and infiltrate the injured tissues and progressively replace tissue-resident macrophages (Brigitte et al., 2010; Louwe et al., 2021). Infiltrating macrophages are classically defined according to their secretome and divided into two subtypes: pro- and anti-inflammatory macrophages. However, macrophage diversity is much more complex due to their great plasticity and could be subdivided into many sub categories (Porcheray et al., 2005; Xue et al., 2014).

A few minutes after the injury, the damage-associated molecular patterns (DAMP) are perceived by the resident macrophages located in the perimysium and epimysium of the muscle, which promote the recruitment of other immune cells such as neutrophils and macrophages derived from monocytes (Brigitte et al., 2010). Ly6C^low^ F4/80^high^ pro-inflammatory macrophages from monocytes invade muscle tissue and then accumulate. They secrete inflammatory cytokines such as IL-6, TNF-α, granulocyte colony-stimulating factor (G-CSF), and IL-1β, and thus eliminate dead cells. They also promote the recruitment of satellite stem cells and the proliferation of myogenic precursors (Li, 2003; Arnold et al., 2007; Serrano et al., 2008; Otis et al., 2014). Ly6C^low^ F4/80^high^ anti-inflammatory macrophages gradually replace, from 48 h post-amputation (hpa), pro-inflammatory macrophages. They secrete pro-resolving cytokines such as insulin-like growth factor 1 (IGF-1), IL-10, and transforming growth factor beta (TGFβ-1), and promote the resolution of inflammation (Arnold et al., 2007; Pelosi et al., 2007; Lu et al., 2011; Mounier et al., 2013; Novak et al., 2014). This switch from the pro- to anti-inflammatory phenotype is possible thanks to the phagocytosis of debris activating signaling pathways such as adenosine monophosphate-activated protein kinase (AMPKa) or CAAT/enhancer binding protein beta (C/EBPb), which in turn promote expression of anti-inflammatory genes (Ruffell et al., 2009; Mounier et al., 2013). Regulatory T cells also participate in this inflammatory switch by inhibiting the release of interferon gamma (IFN-γ) from other pro-inflammatory cells (Panduro et al., 2018). Finally, myogenic mesenchymal cells, such as fibro-/adipogenic progenitors (FAP), are transiently recruited to the site of injury to participate in myogenesis and will then be eliminated by pro-inflammatory macrophages (Lemos et al., 2015). Muscle regeneration takes place via an immune process and a very controlled macrophage response that promotes stem cell differentiation and matrix remodeling (Chazaud, 2020).

#### Macrophages in Zebrafish Regeneration

The zebrafish is able to regenerate many tissues after an injury, such as its caudal fin or its heart, throughout life (Poss et al., 2002; Gemberling et al., 2013; Nguyen-Chi et al., 2015; Hou et al., 2020). The caudal fin fully regenerates via an epimorphic regeneration mechanism that requires the establishment of a transient and highly proliferative structure of undifferentiated cells also called blastema (Lee et al., 2009). Blastema formation allows the complete restoration of certain appendages after amputation by regenerating their mass, structure, and function (Londono et al., 2018; Hou et al., 2020). The heart of the adult zebrafish regenerates via an epimorphic regeneration mechanism associated with a compensatory regeneration mechanism involving the recruitment and proliferation of already differentiated mature cells (Jopling et al., 2010; Gonzalez-Rosa et al., 2011; Sallin et al., 2015). The regeneration of the caudal fin and the heart, although different, both depend on macrophages (Li et al., 2012; Petrie et al., 2014; Xu et al., 2018; Bevan et al., 2020). Zebrafish macrophages appear, as in mammals, during embryogenesis in successive waves from 1 day post-fertilization (dpf) (Herbomel et al., 1999; van der Vaart et al., 2012). They emerge from the lateral plate of the mesoderm and invade the embryo between 12 and 24 h post-fertilization (hpf) via the macrophage colony-stimulating factor (M-CSF). A second wave from hematopoietic stem cells (HSC) appears and reaches the final site at 4 days post-fertilization (dpf). At 3 dpf, macrophages are found in peripheral tissues such as the heart and muscle and accumulated in caudal hematopoietic tissue (CHT) (Herbomel et al., 1999, 2001). The resident macrophages and those derived from monocytes can not be distinguished in zebrafish because of the lack of specific markers. The only study on the subject has focused on the spatial distribution of macrophages (Morales and Allende, 2019). In zebrafish, two macrophage markers are mainly used to follow these cells in real time under microscopy: the gene macrophages expressed 1 (mpeg1) and the microfibrillar associated protein 4 (mfap4) (Ellett et al., 2011). Subtypes of macrophages also exist in zebrafish and, like in mammals, are defined according to their phenotype and functions and referred to as pro- and non-inflammatory subpopulations (Nguyen-Chi et al., 2015, 2017). Once again, this simplified classification does not take into account the great diversity of macrophage subsets that can be further divided into several subtypes in zebrafish as well.

**FIGURE 1:**
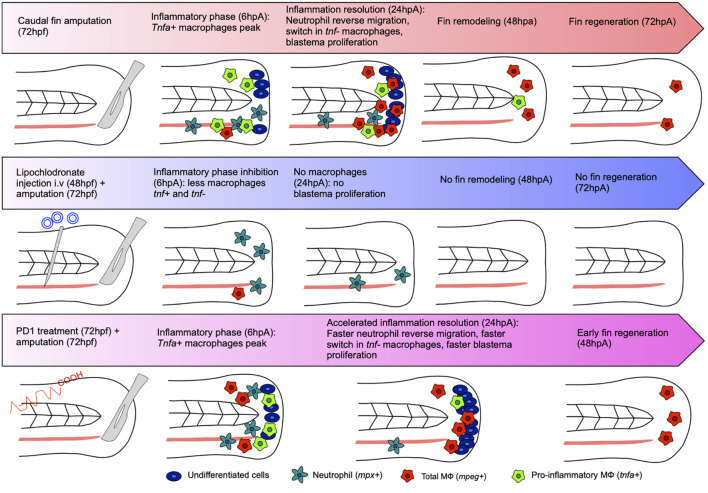
Regeneration and inflammation of the caudal fin under normal, impaired, or improved conditions. Amputation of the caudal fin at 72 hpf leads to an inflammatory phase characterized by the recruitment of neutrophils and macrophages. At 6 hpa, a peak of pro-inflammatory macrophages *tnfa*^+^ appears in the wound area. At 24 hpa, the inflammation is resolved just after the reverse migration of neutrophils. Pro-inflammatory macrophages *tnfa*^+^ are required for blastemal cell proliferation. Then, inflammation resolution occurs characterized by a decreased percentage of *tnfa*^+^ macrophages and a decreased percentage of non-inflammatory *tnfa*^–^ macrophages. At 48 hpa, the morphogenesis of the fin takes place to result in total regrowth of the caudal fin at 72 hpa. Amputation of the caudal fin of 72 hpf zebrafish depleted in macrophages [intravenous injection of lipochlodronate 24 h before amputation (48 hpf)] results in the absence of macrophages at the amputation site 24 hpa, the repression of blastemal cell proliferation, and the absence of caudal fin regeneration at 72 hpa. The treatment of 72 hpf zebrafish larvae with the synthetic drug PD1 after the amputation of their caudal fin results in a normal macrophage response at 6 hpa. However, the inflammation resolves earlier (before 24 hpa) in the larvae treated with PD1 as compared to the untreated control larvae. This was associated with an accelerated (i) reverse migration of neutrophils, (ii) decreased frequency of pro-inflammatory *tnfa*+ macrophages, and (iii) blastema cell proliferation rate in the regenerating caudal fin. Complete restoration of the caudal fin occurred earlier (at 48 hpa) in the zebrafish treated with PD1 as compared to the untreated control that fully regenerate at 72 hpa.

The caudal fin of the zebrafish larva regenerates within 3 days after amputation (Morgan, 1901). A short healing phase appears in the first 6 hpa and then gives way to the formation of an apical epithelial cap (AEC). The AEC established through the secretion of MMP induces the expression of genes allowing the establishment of the blastema, between 12 and 48 hpa. Once the blastema cells are differentiated, they give way to the new member formed (Akimenko et al., 2003; Campbell and Crews, 2008; Lee et al., 2009). Only a few minutes after amputation, macrophages are recruited at the site of the injury and join the few resident tissue macrophages already there. The pro-inflammatory macrophages, *tnfa*^+^, accumulate in the first 24 hpa, then switch their phenotype for some, and disappear for others to give way to the non-inflammatory macrophages, *tnfa*^–^, during the last 48 hpa (Nguyen-Chi et al., 2015, 2017). The sequential depletion of macrophages via the injection of lipochlodronate has also shown the crucial role of these cells. The depletion of early recruited pro-inflammatory macrophages inhibits the proliferation of blastema cells and shows the importance of this early phase in the regenration process. While the depletion of macrophages recruited in the second phase of the regeneration process does not alter the proliferation of blastema cells, it impairs the morphogenesis of the newly formed caudal fin (Nguyen-Chi et al., 2015, 2017; Figure 1). Thus, inflammation is a crucial step for tissue regeneration since its inhibition dramatically impairs the regrowth of the amputated tissue. In this context, we have recently shown that the modulation of the inflammation phase after tissue injury using neuroprotectin/protectin D1 (NPD1/PD1), a proresolving molecule, has direct consequences on the regeneration process. Indeed, PD1 treatment of the regenerating zebrafish larvae after caudal fin amputation accelerates the regrowth process. This pro-regenerative effect induced by PD1 was associated with a rapid resolution of inflammation (Nguyen-Chi et al., 2020; Figure 1).

Conversely, abnormal levels of inflammation have been shown to impair the different phases of zebrafish tissue regeneration. Early inflammation has been characterized by the release of injury-associated factors, neutrophil, and macrophage recruitment (Petrie et al., 2014). In that regard, Hasegawa and colleagues studied the role *il1b* during caudal fin regeneration and found in the cloche mutant (clo), aberrantly overexpressing *il1b*, an excessive and prolonged inflammation that leads to apoptosis and embryonic caudal fin regeneration impairment (Hasegawa et al., 2017). Consistently, anti-inflammatory drug administration rescued the fin regeneration phenotype. They also showed that, in the wild-type fish, *il1b* is mainly produced during the first hours after injury and its expression is quenched after 6 h by macrophages recruited to the wound (Hasegawa et al., 2017). Macrophage tightly regulated inflammatory program, also characterized by TNF-α production, directs the blastema establishment and the regeneration process (Nguyen-Chi et al., 2017). In line with this study, Milkolci and colleagues studied the inflammatory responses upon different caudal fin injuries such as transection and thermal injury. They found that thermal injury induced a stronger neutrophil and macrophage recruitment than a “regular” tissue injury and that among the macrophage subtypes present at the wound site, macrophages expressing *tnfa* were more prevalent during the first 72 h. Interestingly, the healing phase was initiated when the frequency of pro-inflammatory macrophages decreased, i.e., between 24 and 48 h post-injury in the transected tissue or between 48 and 72 h post-injury in the burn wound. This result reveals a 24-h delay in the caudal fin regeneration after thermal injury, which is quite significant given that the whole regeneration process after transection in regular conditions lasted 72 h (Miskolci et al., 2019).

The myocardium of the adult zebrafish heart regenerates within approximately 60 days, after cryoinjury (Chablais et al., 2011; Gonzalez-Rosa et al., 2011). Rapid cardiomyocyte apoptosis first appears in the injured area before an inflammatory phase and the formation of transient fibrosis (Gonzalez-Rosa et al., 2011). Cardiomyocytes already differentiated in the surrounding tissues de-differentiate, then migrate to join and compensate the injured area. A population of undifferentiated cells around the injured area also participates in the formation of the new limb via the establishment of the blastema (Jopling et al., 2010; Sallin et al., 2015; Bevan et al., 2020). Macrophages are recruited few minutes after injury and complete the pool of resident macrophages already present in the tissue (Xu et al., 2018; Bevan et al., 2020). The pro-inflammatory macrophages, *tnfa*^+^, accumulate at 3 days post-cryoinjury (dpc) and induce the formation of a transient fibrosis between 3 and 7 dpc with a deposition of type I collagen. Then, the pro-inflammatory macrophages disappear or switch into non-inflammatory macrophages, *tnf*^–^, at 7 dpa, to allow the resolution of the inflammation and the formation of the new tissue (de Preux Charles et al., 2016; Bevan et al., 2020). Thus, an excessive inflammation also impairs zebrafish heart regeneration. Indeed, Xu and colleagues showed that a mutant deficient for KCNH2, a potassium channel, induced higher levels of inflammatory cytokines after cryoinjury, leading to collagen-rich scars compared to the complete regenerated wild type hearts (Xu et al., 2019). Interestingly, they found that the level of proliferation was higher in the mutants as well as the number of apoptotic cells contributing to thicker scars. Of note, treatment with anti-inflammatory drugs rescued heart regeneration and reduced the fibrotic scar volume (Xu et al., 2019). Xu and colleagues also studied the role of MMP during the early phases of zebrafish heart regeneration. As these proteins are overexpressed after heart resection, they found that MMP at this stage were used as cytokine cleavage activators compared to their role in scar degradation occuring during the late phase of regeneration. Their chemical inhibition was associated with a reduced immune infiltration and heart regeneration impairment showing again a central role of inflammation for tissue regeneration (Simoes et al., 2020).

Altogether, these findings suggest a sequential macrophage subtype activation and/or recruitment, which when dysregulated leads to an impaired or delayed regeneration process. Thus, macrophage inflammatory response plays an active role in the all process either by triggering mesenchymal cells proliferation, apoptosis, or finally tissue morphogenesis and regeneration. Indeed, a well-regulated inflammatory response allows the establishment of the blastema essential for regeneration and tissue morphogenesis. However, when inflammation is inhibited or conversely maintained during the all regeneration process, it leads to the formation of unfunctional fibrotic tissue. Inflammation and its resolution governed by macrophages need to be perfectly coordinated in order to obtain a newly formed tissue, organ, or limb identical to the original one. Hence, identifying mechanisms that control the macrophage response during regeneration is of paramount importance to develop innovative regenerative strategies.

### Role of Macrophages in OA Development and Progression

Synovial macrophages have been shown to be pivotal in the OA vicious circle of cartilage degradation and inflammation (Sambamurthy et al., 2018). Cartilage breakdown products activate macrophages, inducing the secretion of pro-inflammatory cytokines, chemokines, and cartilage-degrading molecules. The synovial membrane undergoes substantial modifications, even before OA joint degradation, that are characterized by the infiltration of immune cells among which are activated macrophages (Hasegawa et al., 2017). The recent description of macrophage phenotypic and functional heterogeneity has raised the hypothesis that an altered orchestration of macrophage response within the synovial membrane might be responsible, in part, of OA development and joint tissue homeostasis impairment.

#### Role of Inflammation in the Development of OA

The role of pro-inflammatory cells in OA has been a matter of debate only over the past decade. The pathogenesis of OA was initially associated just with mechanical stress leading to articular cartilage erosion and pathological bone growth (Dequeker and Luyten, 2008). Even though the levels of pro-inflammatory cytokines are not as pronounced compared to other pro-inflammatory joint diseases, such as rheumatoid arthritis (RA) (Hampel et al., 2013), new evidence highlights the role of inflammation in the pathogenesis of OA.

As mentioned above, tissue injury triggers several steps including an inflammatory phase, cell proliferation, tissue remodeling, and a resolution phase. The role of inflammation during tissue injury is key to initiate repair mechanisms; however, if the resolution phase is not achieved in a specific time window, neither reparation nor regeneration will occur (Ellis et al., 2018). OA associated with trauma, microtrauma, or normal aging, chondrocytes, and extracellular matrix begin to disintegrate, generating DAMPs by resident cells, including resident macrophages, initiating the inflammatory response (Foell et al., 2007; van Lent et al., 2012; Orlowsky and Kraus, 2015). As a result of the activation of inflammatory signaling pathways, cells release high levels of pro-inflammatory cytokines, such as IL-6, IL-1β, and TNF-α (Kany et al., 2019), and chemokines such as CCL2 (Raghu et al., 2017) are key to attract circulating monocytes and other blood cells to site of injury. Then, after DAMPs-mediated activation, the recruited cells will secrete more soluble mediators, triggering a positive feedback loop (Lambert et al., 2020). When the acute injury is under control, macrophages play a key role by phagocytizing cellular debris and secreting anti-inflammatory cytokines, mediators, and growth factors that promote wound repair (Vannella and Wynn, 2017). Conversely, during OA the absence of a resolution phase leads to chronic inflammation, which contributes to tissue damage. However, it is still unknown why the resolution phase is not achieved.

#### Resident Synovial Macrophages in the Joint

Resident macrophages can be present in a wide variety of tissues, particularly in the joint, and they can be found in the synovium, adipose tissue, subchondral bone, muscle, ligaments, and tendons (Wu et al., 2020). Increasing evidence has shown that subsets of resident macrophages with different phenotypes play a pivotal role in anabolic and catabolic aspects during the progression of OA. The synovial tissue is composed of the lining layer, where macrophages and fibroblasts can be found and is in direct contact with the synovial fluid, and the sublining layer made by small blood vessels, lymphatic vessels, fibroblast, and also macrophages (Wu et al., 2020).

**FIGURE 2:**
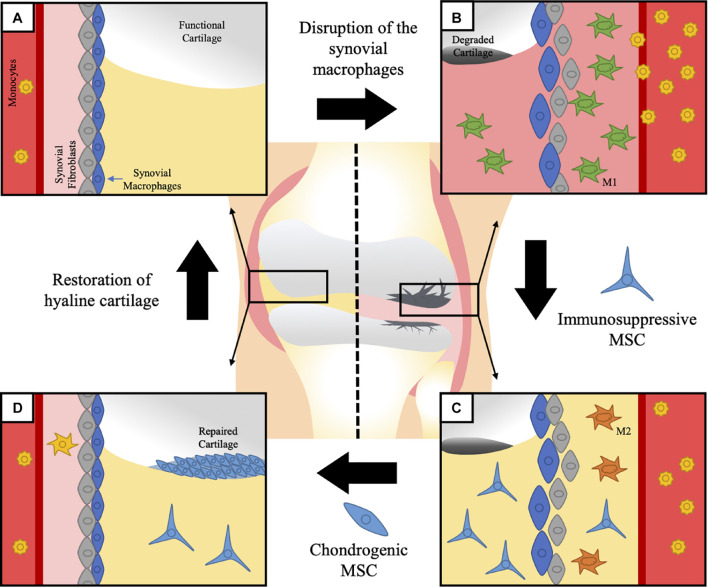
Macrophages in generation of OA and MSC therapeutic potential. Synovial tissue resident macrophages (STMs) and monocyte-derived macrophages have been suggested to have an active role on the pathophysiology of OA. Although the direct participation of STM on OA has not been shown yet, their physiological function opens a clear path between the origin of the disease and its progression. **(A)** STMs help maintain the regular homeostasis of the articular cartilage and the synovial cavity, while they keep a physical barrier separating the intra-articular space from the sublining fibroblasts. Once this barrier is disrupted, local inflammation of the joint is triggered. **(B)** The degradation of the articular cartilage is associated to an increase in the infiltration of pro-inflammatory monocyte-derived macrophages and disintegration of the normal synovial structure. The local inflammation impairs the already limited ability of the cartilage to regenerate itself, therefore leading to irreversible and progressive damage. Current therapies have failed to present a united front covering both fronts in the fight against OA: control of the immune response and functional regeneration of the cartilage. **(C)** To this end, MSC present an amazing therapeutic tool, due to their know capacity to control macrophages and induce the switch from pro-inflammatory macrophages, to anti-inflammatory macrophages. Multiple strategies have been suggested nowadays to activate and enhance the immunomodulatory potential of MSC previous to their application in the affected joints, which could present a relevant improvement to accomplish the first objective. **(D)** The immunoregulatory environment generated with an initial application of MSC would facilitate a secondary step to repair the lost tissue with MSC directed to differentiate into hyaline cartilage and an eventual restoration of normal structure and function of the osteoarthritic joint.

Murine macrophages expressing the chemokine receptor CX_3_CR1 form a dense physical barrier at the border of the lining layer, separating the intra-articular space from the sublining layer where CX_3_CR1^–^ interstitial macrophages are present (Culemann et al., 2019; Figure 2). Single-cell RNA sequencing analysis showed that these CX_3_CR1^+^ macrophages express several immunoregulatory-related genes, while CX_3_CR1^–^ macrophages can be divided in several subpopulations, evidencing a high degree of heterogeneity (Culemann et al., 2019). After the induction of serum-transfer arthritis (STA) and collagen-induced arthritis (CIA), CX_3_CR1^+^ lining macrophages respond by changing their morphology and spatial orientation but without proliferating, while CX_3_CR1^–^ MHC-II^+^ sublining macrophages rapidly proliferate. Moreover, the authors shown that CX_3_CR1^–^ MHC-II^+^ macrophages can further differentiate not only to CX_3_CR1^+^ lining macrophages but also to interstitial macrophages expressing resistin-like molecule (RELM)-α, another subset related to immunosuppressive functions (Batugedara et al., 2018; Culemann et al., 2019).

Rheumatoid arthritis (RA) is another inflammatory-joint disease that can give us more information about macrophage subset functions in joint disorders. RNA sequence analysis of human synovial tissue macrophages (STMs) from healthy donors and patients in RA sustained remission revealed that most of them are MerTK^+^CD206^+^; both of them are markers of healthy immune-homeostatic STM (Kurowska-Stolarska and Alivernini, 2017). These STM are present in the lining layer; they produce lipid mediators and induce an *in vitro* reparatory response in fibroblast through increasing the expression of TGF-β and collagen-related genes (Alivernini et al., 2020). In contrast, STM from RA patients display a MerTK^–^CD206^–^ profile, they are present in the sublining layer and produce pro-inflammatory cytokines and alarmins. They induce pro-inflammatory responses when cocultured with fibroblasts through the upregulation of IL-6 and CCL2 and also produce mediators of cartilage and bone degradation such as MMP1/3 and receptor activator of nuclear κB ligand (RANKL), respectively (Alivernini et al., 2020). Currently, there is no similar study reporting similar observations in OA. Therefore, studies on the interaction between different subsets of tissue resident macrophages and other cell types present in the joint niche including synovial fibroblasts and chondrocytes could reveal important aspects of the onset and outcome of OA pivotal for early OA diagnosis and the development of innovative therapies.

#### Pro-inflammatory/Anti-inflammatory Macrophage Phenotypes in OA

The pro- and anti-inflammatory categorization of macrophages is based on their response to *in vitro* stimulation. However, this classification is recognized as the edges of a broad phenotypic spectrum of macrophage subsets (Martinez and Gordon, 2014; Xue et al., 2014), which is more congruent with findings in *in vivo* models or patient samples. Based on the expression of CD11c and CD86 as pro-inflammatory macrophage markers, and CD206 and CD163 as anti-inflammatory macrophage markers, Liu et al. (2018) showed that a higher pro-inflammatory/anti-inflammatory macrophage ratio in the synovial fluid and peripheral blood of OA patients is directly correlated with the severity of the disease and may contribute to OA progression (Figure 2).

Folate receptor (FR)-β has been reported to be present in macrophages producing pro-inflammatory cytokines (Nagayoshi et al., 2005) and to be a useful marker to trace macrophages in OA. By using Etarfolatide, a folate receptor-specific molecular imaging agent, Kraus et al. identified for the first time the presence of activated macrophages in synovial tissue of OA patients *in vivo* using SPECT-CT imaging (Kraus et al., 2016). In this study, the presence of activated macrophages was directly correlated with the severity of the symptoms. Immunostaining of joint fluid showed the coexpression of the pro-inflammatory macrophage molecule called inducible nitric oxide synthase (iNOS) and the anti-inflammatory macrophage marker TGF-β in these activated macrophages (Kraus et al., 2016), which was consistent with previous studies showing the presence of FR-β^+^CD163^+^ macrophages in the lining layer of OA patients, also exhibiting a mixed pattern of pro-inflammatory/anti-inflammatory macrophage markers (Kraus et al., 2016). Moreover, RNA-sequencing analysis of the synovial tissue of OA patients showed a mixed of macrophage subtypes (Wood et al., 2019). Indeed, the authors identified two different subsets of macrophages: classic OA (cOA) macrophages and inflammatory-like OA (iOA) macrophages. These cOA macrophages expressed cartilage remodeling-related genes such as *HTRA1*, which can modulate synovial fibroblast to produce MMPs, and *EFEMP1*, which can potentially act as a negative regulator of chondrogenesis. On the other hand, iOA macrophages displayed a strong proliferation signature overexpressing *MKI67*, which encode the Ki67 protein associated to cell proliferation, and *E2F8* and *CDT1*, which can modulate cell proliferation (Wood et al., 2019). This study paves the way for the stratification of OA patients that will allow the development of personalized disease-modifying treatments and/or the identification of new therapeutic targets.

### MSC as a Therapeutic Strategy to Target Macrophages in OA

MSC have been intensively studied as a potential tool in the treatment of OA to trigger both joint tissue repair and pathogenic immune response through their proregenerative and anti-inflammatory properties (Zhang et al., 2019). As discussed previously, clinical trials using MSC for OA treatment have shown that MSCs are effective in relieving pain and improving functionality in OA patients (Song et al., 2020). In particular, autologous adipose-tissue-derived MSC (AD-MSC) have been shown to decrease the size of cartilage defect in OA patients, while the volume of cartilage increased in the medial femoral and tibial condyles, with a hyaline-like cartilage regeneration 6 months after the intra-articular injection of MSC (Jo et al., 2014). Conversely, some studies have suggested that joint-resident MSC might be involved in the development of the disease. Indeed, in adult human cartilage, the increased frequency of CD166^+^ MSC-like progenitors during OA was reported. These cells exhibit significantly higher expression of COL10A1 and RUNX2, which are specific markers of hypertrophic OA cartilage, suggesting that the tissue-resident MSC of OA patients are pathogenic, making the use of autologous MSC more troublesome (Jayasuriya et al., 2018). This pathogenic response of MSC has been associated to the inflammatory microenvironment of the diseased joint. The multiple pro-inflammatory cytokines present in the synovial fluid (SF) during OA, such as IFN-γ and TNF-α, have been demonstrated to impair the production of glycosaminoglycans, in equine bone-marrow-derived MSC (BM-MSC) and SF-derived MSC (SF-MSC) under chondrogenic differentiation. Specifically, pro-inflammatory cytokines induce a reduction in the expression of SOX-9, TGF-β1, aggrecan, and collagen II in BM-MSC, whereas in SF-MSC, they only reduce the levels of aggrecan (Zayed et al., 2016). Therefore, these results and others suggest that the local inflammation encountered in the joint during OA might be deleterious for MSC multipotency including their chondrogenic potential and tissue repair (van der Kraan, 2019). Therefore, studies using MSC for OA treatment have considered their immunoregulatory and anti-inflammatory properties as a perfect combination to treat OA defects (Figure 2).

The immunomodulatory capabilities of MSC have been widely described, being able to inhibit pro-inflammatory cells from the innate and adaptive immune system and simultaneously favor an anti-inflammatory environment (Contreras et al., 2016). More precisely in the context of OA, MSC have been shown to enable a phenotypic switch of pro-inflammatory macrophages/monocytes to anti-inflammatory subsets (Abumaree et al., 2013; Pers et al., 2018). MSCs exert this immunosuppressive function through a wide variety of molecular mechanisms, such as the release of soluble factors including TNF-α-stimulated gene/protein 6 (TSG-6) (Song W.J. et al., 2017) or prostaglandin E2 (PGE2) (Vasandan et al., 2016). Moreover, cell-to-cell contact has been reported to be involved in the mechanisms mediating MSC immunoregulatory properties in particular by enhancing the production of TSG-6 by MSC, through CD200/CD200R1 receptor complex interaction (Li et al., 2019). These molecules have been described to induce the conversion of TNF-α- and IL-1β-producing pro-inflammatory macrophages into IL-10-producing anti-inflammatory cells and a subsequent decrease in joint inflammation and enhancement of cartilage regeneration (Harrell et al., 2019b). MSC-derived TSG-6 has a known interaction with the CD44 receptor on macrophages. This interaction inhibits TLR2-mediated translocation of nuclear factor kappa κβ (NF-κβ) to the nucleus alleviating secretion of inflammatory mediators (Choi et al., 2011). PGE2 is a small molecule derived from the metabolism of arachidonic acid that is produced by the inducible enzyme cyclooxygenase-2 (COX2) (Kalinski, 2012). MSC-derived PGE2 binds to EP4 receptors on macrophages and promotes the production of IL-10, through a cAMP-dependent pathway (Na et al., 2015). Interestingly, this immunoregulatory capacity of MSC has been widely described to be triggered upon stimulation with an inflammatory environment. Cytokines such as IFN-γ and TNF-α have been shown to induce the expression of TSG-6 and PGE2, as well as many other anti-inflammatory mediators produced by MSC (Contreras et al., 2016; Saldana et al., 2019). Therefore, although deleterious for the chondrogenic differentiation of MSC, this inflammatory environment, in part characteristic of the synovial fluids derived from OA patients (Mabey et al., 2016; Yang et al., 2016; Domenis et al., 2017; Liao et al., 2018), is fundamental for the activation of their immunomodulatory properties (Ren et al., 2010; Figure 2).

Another interesting mechanism by which MSC protect the injured joint tissues is their secreted extracellular vesicles (EVs). MSC-derived EVs (MSC-Evs) can contain the same immunosuppressive mediators that have been already mentioned for their parental cells but can also can transport molecules that cannot be secreted including other proteins, enzymes, organelles, lipids, metabolites, nucleic acids, and non-coding RNAs (Harrell et al., 2019a). MSC-EVs have been reported to exert similar therapeutic effects as their parental cells without their disadvantages, making the use of MSC-EVs an interesting cell-free therapeutic strategy. Indeed, they inhibit the polarization of macrophages toward a pro-inflammatory phenotype and promote the generation of anti-inflammatory cells in multiple experimental models of diseases (Wang et al., 2020). In the context of OA, MSC-EVs have been reported to promote anti-inflammatory macrophage infiltration in OA synovial membrane and reduce the frequency of pro-inflammatory cells. This was associated with a decreased level of IL-1β and TNF-α and an increased proliferation of chondrocytes and synthesis of the extracellular matrix (Zhang S. et al., 2018). Additionally, the pre-conditioning of MSC might increase the therapeutic effect of MSC-EVs. Upon stimulation with lipopolysaccharide, MSC-EVs have been reported to have a more significant regulatory effect on macrophage polarization, through the overexpression of the micro-RNA let-7b (Ti et al., 2015). Similarly, IL-1β has been described to stimulate the accumulation of miR-146a in human umbilical cord-derived MSC-EVs, which promotes the transition of pro-inflammatory macrophages toward an anti-inflammatory phenotype (Song Y. et al., 2017). Several other strategies have been proposed to enhance MSC properties. Among them, miRNAs overexpression, hypoxia pre-conditioning, or metabolic reprogramming have been successfully investigated (Zhao X. et al., 2020; Zhao Y. et al., 2020; Contreras-Lopez et al., 2021); however, they have not been yet explored in the context of experimental OA.

## Conclusion/Perspectives

OA is a multifactorial joint disease, involving synovial tissue, cartilage, and subchondral bone. The physiopathology of OA is complex and combines chronic synovial inflammation and accumulation of senescent cells, associated with dedifferentiation and hypertrophy of chondrocytes and subchondral bone remodeling. Thus, identifying a new therapeutic target and developing innovative OA therapy is challenging. Here, we reviewed the interactions between macrophages and MSC and remind the critical impact between inflammation and regenerative process through EV and TSG-6. This dialogue is possible through cytokines and chemokines release and EV delivering of proteases and nucleotides. Synovial macrophages have a dual role of macrophages in damaging the cartilage and in repair and regeneration of the tissue in a second step. The identification of the mechanisms underlying the macrophage phenotypic switch during tissue regeneration is of major importance in the field of regenerative medicine and will offer new therapeutic strategies. Targeting the upstream mechanisms of inflammation will not only limit joint tissue degradation but also promote progenitor cells to differentiate and regenerate the damaged tissues.

## Author Contributions

All authors wrote the manuscript, contributed to the article, and approved the submitted version.

## Conflict of Interest

CT-A and MW were employed by CellVax. The remaining authors declare that the research was conducted in the absence of any commercial or financial relationships that could be construed as a potential conflict of interest.

## Publisher’s Note

All claims expressed in this article are solely those of the authors and do not necessarily represent those of their affiliated organizations, or those of the publisher, the editors and the reviewers. Any product that may be evaluated in this article, or claim that may be made by its manufacturer, is not guaranteed or endorsed by the publisher.
